# Identification of Survival-Associated Hub Genes in Pancreatic Adenocarcinoma Based on WGCNA

**DOI:** 10.3389/fgene.2021.814798

**Published:** 2022-01-03

**Authors:** Liya Huang, Ting Ye, Jingjing Wang, Xiaojing Gu, Ruiting Ma, Lulu Sheng, Binwu Ma

**Affiliations:** ^1^ Department of Gastroenterology, The General Hospital of NingXia Medical University, Yinchuan, China; ^2^ Department of Emergency Medicine, Shanghai Jiao Tong University Affiliated Sixth People’s Hospital, Shanghai, China; ^3^ Department of Neurology, The General Hospital of NingXia Medical University, Yinchuan, China

**Keywords:** WGCNA, gene module, network construction, functional regulator, pancreatic adenocarcinoma

## Abstract

Pancreatic adenocarcinoma is one of the leading causes of cancer-related death worldwide. Since little clinical symptoms were shown in the early period of pancreatic adenocarcinoma, most patients were found to carry metastases when diagnosis. The lack of effective diagnosis biomarkers and therapeutic targets makes pancreatic adenocarcinoma difficult to screen and cure. The fundamental problem is we know very little about the regulatory mechanisms during carcinogenesis. Here, we employed weighted gene co-expression network analysis (WGCNA) to build gene interaction network using expression profile of pancreatic adenocarcinoma from The Cancer Genome Atlas (TCGA). STRING was used for the construction and visualization of biological networks. A total of 22 modules were detected in the network, among which yellow and pink modules showed the most significant associations with pancreatic adenocarcinoma. Dozens of new genes including PKMYT1, WDHD1, ASF1B, and RAD18 were identified. Further survival analysis yielded their valuable effects on the diagnosis and treatment of pancreatic adenocarcinoma. Our study pioneered network-based algorithm in the application of tumor etiology and discovered several promising regulators for pancreatic adenocarcinoma detection and therapy.

## Introduction

Pancreatic cancer ranks the 7th leading cause of cancer mortality worldwide with increasing incidence and poor outcomes (McGuigan et al., 2018). According to the SEER registry, 60,430 new cases and 48,220 deaths of pancreatic cancer have been estimated in the United States in 2021 (Siegel et al., 2021). And pancreatic cancer is predicted to rise from being the 4th to the 2nd most common cause of cancer-related death in the United States by 2030 (Rahib et al., 2014). Pancreatic adenocarcinoma and its variant are the most prevalent subtype of pancreatic cancer and attributed to approximately 90% of all cases (Feldmann et al., 2007). Pancreatic intraepithelial neoplasia, intraductal papillary mucinous neoplasms and mucinous cystic neoplasms are the best characterized precursors of this cancer (Esposito et al., 2014). Due to the insidious nature of pancreatic adenocarcinoma, most patients have already carried metastases such as node upon diagnosis, resulting in 5-years relative survival of about 10% (Luchini et al., 2016; Siegel et al., 2021). Although great efforts including surgical resection, adjuvant chemotherapy and serum biomarker CA19-9 have been made in early detection and treatment of pancreatic adenocarcinoma, medical limitations still exist because of low sensitivity and high expenses. Recently, several driver genes including KRAS, CDKN2A, TP53 and SMAD4 have been identified from an evolutionary perspective (Vogelstein and Kinzler, 2015; Makohon-Moore and Iacobuzio-Donahue, 2016). Another study has explored novel immune-related gene signatures in pancreatic adenocarcinoma (Chen B. et al., 2021). However, the above evidence is far from adequate to provide therapeutic targets as multiple initiating events embedded with genes were undiscovered in pancreatic adenocarcinoma. Hence, it is imperative to identify new risk genes and their regulatory network in order to elucidate pancreas carcinogenetic mechanisms as well as guide researchers to develop new therapeutic strategies.

As a systematic method used in oncology research that aims at finding co-expressed genes through calculating gene connectivity, weighted gene co-expression network analysis (WGCNA) can analyze the relationship between modules and specific traits followed by clustering genes and forming modules (Langfelder and Horvath, 2008; Chang et al., 2013; Yang et al., 2018). It is widely used in exploring functionality of the whole transcriptome for its particularly powerful computing capability (Zhou et al., 2018). In this study, we employed WGCNA to build a gene interaction network using the expression profile of pancreatic adenocarcinoma from The Cancer Genome Atlas (TCGA). A total of 22 modules were detected in the network, among which yellow and pink modules showed the most significant associations with pancreatic adenocarcinoma. Dozens of new genes including PKMYT1, WDHD1, ASF1B, and RAD18 were identified. Further survival analysis yielded their valuable effects on the progression of pancreatic adenocarcinoma.

## Methods

### Pancreatic Adenocarcinoma RNA-Sequencing Datasets

The RNA-sequencing data of 175 pancreatic adenocarcinoma patients was downloaded from the TCGA database (https://portal.gdc.cancer.gov/). As previously described, the gene expression levels were quantified as FPKM (fragments per kilobase per million mapped reads) using TopHat and HTSeq-count (Kim et al., 2013; Anders et al., 2015). The TCGA sample information was listed in Supplementary Material S1. Within the 175 tumor samples, 2 samples did not have stage information, 7 samples were in stage I, 24 samples were in stage II, 139 samples were in stage III, and 3 samples were in stage IV. The sample distribution also stressed the importance of identification of new genes for diagnosis and therapy.

### Co-Expression Network Construction

R package WGCNA was used for hub genes screening and co-expression of gene pair detection. Elements in the gene co-expression matrix were the weighted values of correlation coefficient between gene pairs. The soft-thresholding function was applied to calculate the power parameter. Dynamic tree cut method was utilized to identify co-expressed gene modules and a dendrogram of genes was produced via a hierarchical clustering approach based on dissimilarity of the unsigned topological overlap matrix (TOM). Finally, genes with similar expression profiles were grouped into network modules.

### Enrichment Analysis of Module Genes

R package clusterProfiler was used to perform functional enrichment analysis on clustered genes in pink and yellow modules. A hypergeometric distribution test was applied to detect enrichment terms, and *p* values were adjusted by false discovery rate (FDR) method with a cutoff FDR <0.05 (Yu et al., 2012).

### Visualization of Gene Networks

Construction and analysis of networks were carried out using STRING (11.0) (Szklarczyk et al., 2019).

### The Survival Analysis of Hub Genes

There were 175 pancreatic adenocarcinoma patients with the overall, disease free survival time (months) and the survival status. We performed survival analysis using the Cox proportional hazard regression model on these samples (Andersen and Gill, 1982). For each gene, the patients were divided into two groups: the patients with expression levels smaller than the median and the patients with expression levels greater than or equal to the median. The Kaplan-Meier plot was used to describe the survival curves of these two groups of patients. The significance of the survival difference between these two patient groups was evaluated by the log-rank test *p* value. If the *p* value was less than 0.05, its survival was considered as significantly different. The R package survival (https://CRAN.R-project.org/package=survival) was used to perform the survival analysis.

## Results and Discussion

### Construction of Co-Expression Network

**FIGURE 1:**
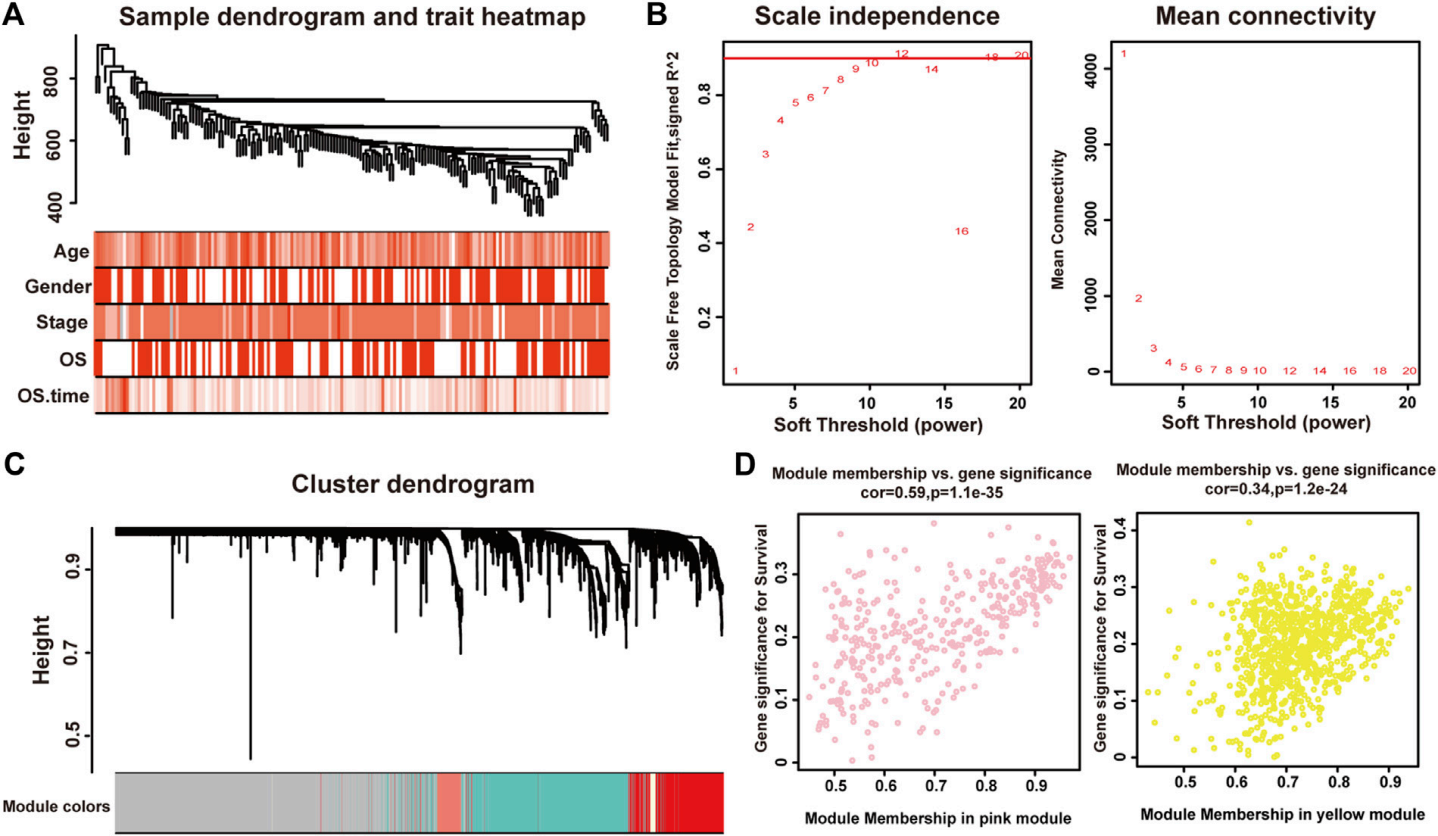
Identification of modules associated with the clinical traits of pancreatic adenocarcinoma. **(A)** Clustering dendrogram of 175 samples. **(B)** The relationship between soft threshold (power) and network properties. Left panel: The relationship between soft-threshold (power) and scale-free topology. Right panel: The relationship between soft threshold (power) and mean connectivity. We set the soft threshold (power) to be twelve to satisfy scale-free topology of the network. **(C)** Total genes were clustered in 22 modules. Each module was marked with one color. **(D)** A scatterplot of Gene Significance (GS) for disease vs. Module Membership (MM) in the pink (left panel) and yellow (right panel) modules.

We used the Pearson’s correlation coefficient to cluster the samples in TCGA. After removing outliers, we drew a sample clustering tree (Figure 1A). The weighted gene co-expression network was constructed from 60,483 genes through WGCNA approach. Here, soft-thresholding power was set to be twelve to satisfy scale-free topology of the network (Figure 1B), in which *R* ^2^ was used to check how well the network fit the scale freeness. And we detected 22 modules in this network, whose relationship was shown in a cluster dendrogram (Figure 1C). The number of members in different modules varied widely. Besides the grey module comprised of many un-classified members, turquoise module contained the maximum 1,508 genes, while the minimum 36 genes were included in darkred module.

Each module represented a group of genes with similar expression profiles across samples. Next, we quantified module-trait associations (Supplementary Material S2), among which the pink and yellow modules showed the most significant associations with pancreatic adenocarcinoma. The corresponding correlation coefficients of pink and yellow modules were 0.3 (*P* = 4 × 10^−5^) and −0.28 (*P* = 2 × 10^−4^), respectively. Clearly, Gene Significance (GS) and Module Membership (MM) analysis illustrated that genes highly significantly associated with pancreatic adenocarcinoma were also the most important elements of modules associated with pancreatic adenocarcinoma (Figure 1D).

### Enrichment Analysis of Module Genes

**FIGURE 2:**
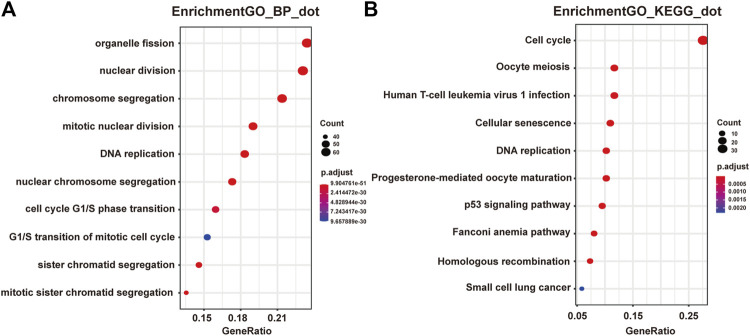
Functional enrichment analysis of genes in the pink module. **(A)** GO analysis showed the top 10 enriched biological processes in the pink modules. **(B)** KEGG analysis showed the top 10 enriched pathways in the pink modules.

Next we performed Gene Ontology (GO) and Kyoto Encyclopedia of Genes and Genomes (KEGG) enrichment analysis of these two modules. As presented in Figures 2A,B, genes in the pink module were significantly enriched in cell cycle, DNA replication, nuclear division, and regulation of cell cycle phase transition with adjusted *p* value smaller than 0.05, while genes in the yellow module were not enriched in any terms or pathways (data not shown), conferring the importance of these biological functions on pancreatic adenocarcinoma development. And genes in the pink module were selected for the following functional analysis.

### Identification of Hub Genes

**FIGURE 3:**
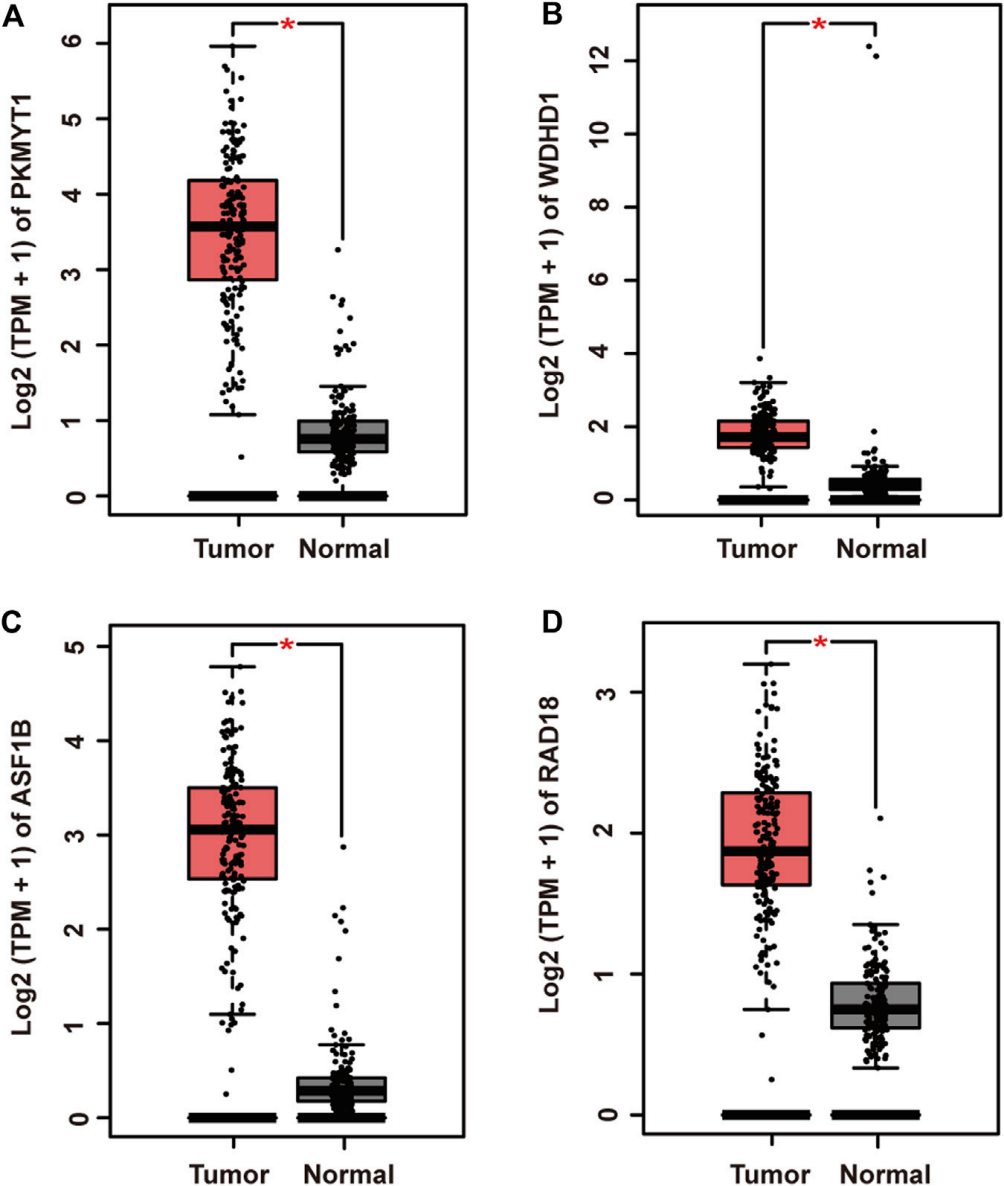
Relative mRNA expression of four hub genes in pancreatic adenocarcinoma and adjacent normal tissues from TCGA. **(A)** PKMYT1. **(B)** WDHD1. **(C)** ASF1B. **(D)** RAD18. The expressions of these genes were significantly up-regulated in tumor samples.

To further elucidate gene regulatory relationship in the module, we constructed co-expressed gene networks in the pink module (Supplementary Material S3) and identified master regulators with most connections with others. Finally, PKMYT1, WDHD1, ASF1B, and RAD18 stood out in the network. Next, we investigated their expression patterns in pancreatic adenocarcinoma. As expected, PKMYT1 (Figure 3A), WDHD1 (Figure 3B), ASF1B (Figure 3C), and RAD18 (Figure 3D) were significantly up-regulated in tumor tissues compared to adjacent normal tissues from TCGA, emphasizing their promising roles in carcinogenesis.

### Functional Analysis of Survival-Associated Key Genes

**FIGURE 4:**
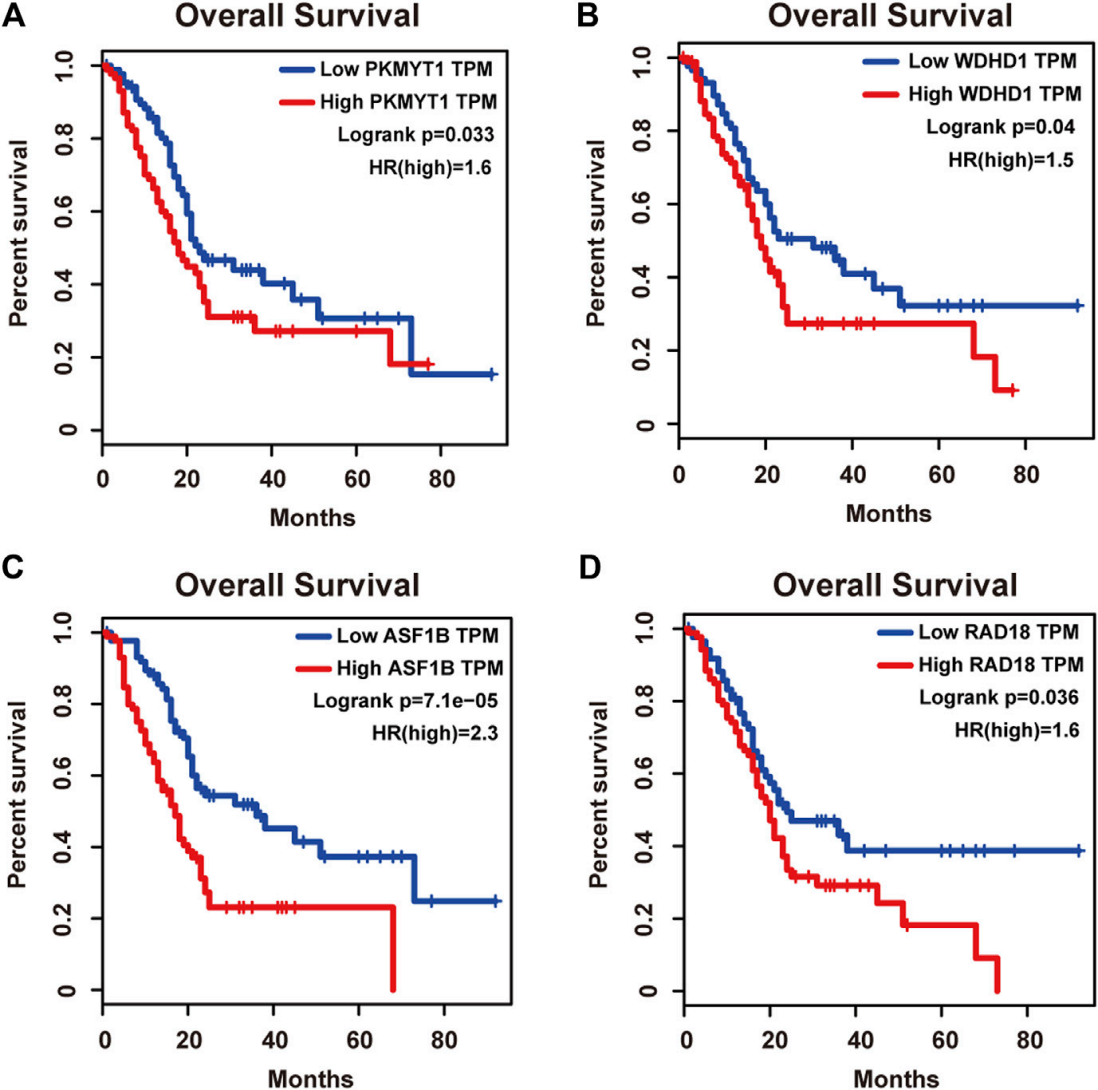
Overall survival analysis of the four hub genes in pancreatic adenocarcinoma based on the Kaplan-Meier plotter. **(A)** PKMYT1. **(B)** WDHD1. **(C)** ASF1B. **(D)** RAD18. The high expressions of these four genes were associated with high risk.

**FIGURE 5:**
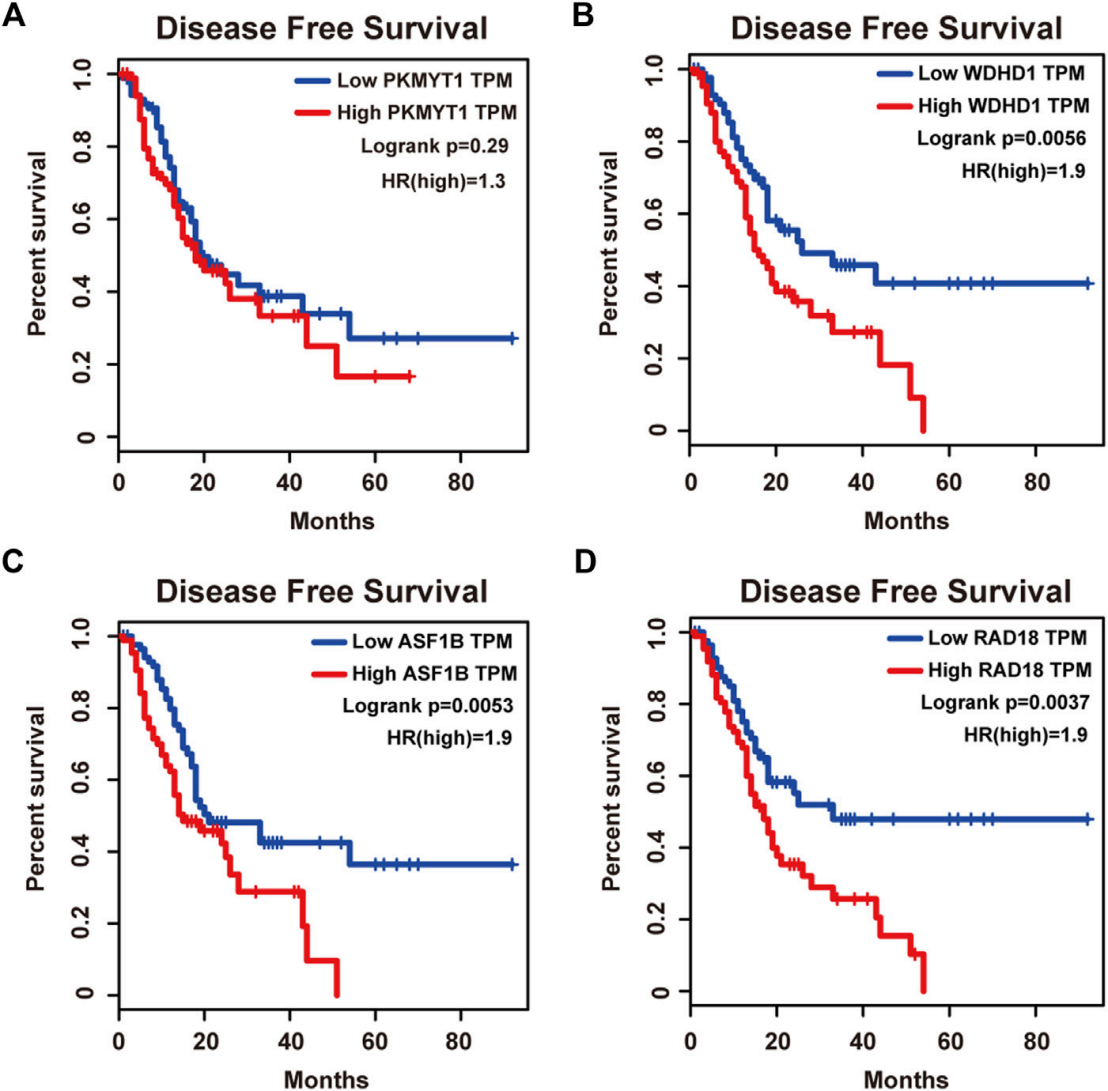
Disease free survival analysis of the four hub genes in pancreatic adenocarcinoma based on the Kaplan-Meier plotter. **(A)** PKMYT1. **(B)** WDHD1. **(C)** ASF1B. **(D)** RAD18. The high expressions of WDHD1, ASF1B and RAD18 were associated with high risk.

Protein kinase, membrane associated tyrosine/threonine (PKMYT1), also known as MYT1, is a member of the WEE1 family of protein kinases, exerting key effects on Golgi and endoplasmic reticulum assembly (Chen et al., 2020). PKMYT1 was firstly recognized as a kinase capable of phosphorylating Cdc2 at Thr14 and Tyr15 (Mueller et al., 1995). Increasing studies have revealed its negative roles in cell cycle progression through suppressing cell cycle-associated factors, such as Cyclin A and CDK1 (Varadarajan et al., 2016), leading to its promising relationship with cancer. Previous studies have shown that PKMYT1 promoted cell proliferation and apoptosis resistance in multiple cancers, such as esophageal squamous cell carcinoma (Zhang et al., 2019), non-small cell lung cancer (Sun et al., 2019), prostate cancer (Wang et al., 2020), hepatocellular carcinoma (Liu et al., 2017), and gastric cancer (Zhang et al., 2020). Also overexpression of PKMYT1 predicted unfavorable prognosis in breast cancer (Liu Y. et al., 2020) and clear cell renal cell carcinoma (Chen et al., 2020), implicating it as an appealing therapeutic target. Nevertheless, an understanding of the correlation between PKMYT1 and pancreatic adenocarcinoma remains elusive, and our Kaplan-Meier analysis indicated, for the first time, better overall survival in the low transcription group approaching significance (Figure 4A), consistent with its tumor promotion roles in other cancers. However, disease free survival time did not show difference between low and high expression groups of PKMYT1 (Figure 5A). Through our network-based analysis, we also emphasized its crucial roles in the regulation of cell cycle (Figure 2A), which provided experimental clues for further investigations.

WD repeat and high-mobility group box DNA-binding protein 1 (WDHD1), an orthologue of Ctf4 in budding yeast and Mcl1 in fission yeast, is a DNA-binding protein involved in DNA replication and cell cycle (Abe et al., 2018). Recent studies have observed the overexpression of WDHD1 in the great majority of lung cancers and esophageal squamous cell carcinomas (Sato et al., 2010). WDHD1 has also been reported to facilitate the abrogation of G1 checkpoint upon DNA damage, leading to genomic instability and eventually tumorigenesis (Zhou et al., 2020). Moreover, WDHD1 could accelerate cell proliferation, cell viability, and metastasis in several cancers including cholangiocarcinoma and breast cancer (Sato et al., 2010; Liu et al., 2019; Ertay et al., 2020; Zhou and Chen, 2021). In accordance with the above research, both our overall and disease free survival analysis exhibited that high levels of WDHD1 correlated with poor patient outcome (Figures 4B, 5B), confirming the oncogenic function of WDHD1 in pancreatic adenocarcinoma and expanding it roles in cancer biology which need further validations.

As one of histone H3-H4 chaperone anti-silencing function 1 (ASF1) isoforms, ASF1B plays important roles in chromatin-based progression of cellular DNA replication and transcription regulation, especially in cell proliferation (Paul et al., 2016). Accumulating evidence has shown that up-regulation of ASF1B stimulated cancer cell proliferation, DNA replication and migration, accompanied by restrained cell cycle arrest and apoptosis (Misiewicz-Krzeminska et al., 2013; Han et al., 2018; Zhou et al., 2019; Liu X. et al., 2020; Zhang et al., 2021), which was also consistent with our enrichment analysis of module genes (Figure 2A). In addition, several studies have underlined the important prognostic impact of ASF1B on a variety of cancers (Corpet et al., 2011; Chen Z. et al., 2021; Feng et al., 2021). Another exciting finding was that ASF1B could regulate immune infiltration through affecting immune-related genes and pathways such as antigen processing and presentation and natural killer cell-mediated cytotoxicity (Hu et al., 2021; Zhan et al., 2021), conferring its strong hints on potential immunotherapeutic target for several malignancies. The both overall and disease free survival analysis also demonstrated that the high expression of ASF1B was associated with high risk (Figures 4C, 5C), suggesting it serving as a tumor promoter in pancreatic adenocarcinoma.

RAD18 is an E3 ubiquitin ligase best known for its key roles in the monoubiquitylation of proliferating cell nuclear antigen (PCNA) in response to stalled replication forks, thus initiating DNA damage repair signaling (Williams et al., 2011; Kanu et al., 2016; Yang et al., 2017). Increasing reports have shown that RAD18 enhanced motility and invasiveness of cancerous cells, evidenced by the positive correlations between RAD18 and vital mediators of cell invasion and proliferation such as MMP-1 and MMP-9 (Zou et al., 2018; Xie et al., 2019). Meanwhile, a recent study has found elevated RAD18 was associated with gastric cancer progression and poor prognosis (Baatar et al., 2020), considering it as a novel prognostic biomarker. Accordingly, both overall and disease free survival time was significantly higher in patients with low RAD18 expression, compared with the high RAD18 expression group (Figures 4D, 5D), highlighting its potential values in the treatment and prognosis of pancreatic adenocarcinoma.

## Conclusion

Gene correlation approaches provide preliminary steps toward genetic interaction networks and offer clues about the function of unknown genes. Here, we employed WGCNA to identify novel hub genes including PKMYT1, WDHD1, ASF1B, and RAD18, and proposed for the first time their oncogenic roles during pancreatic adenocarcinoma progression. Further survival analysis verified their effective roles in predicting prognosis. In-depth mechanisms explaining their ability to allow neoplastic cells to breach tumorigenic barriers are needed. Meanwhile, we should not ignore the limitations of WGCNA as it is based on transcriptomic data and insufficient to reflect cell status globally, in which multilayer data including mutations, copy number variations and proteomic data is also needed to be taken into account for bettering understanding mechanisms triggering cancer. Also, stronger computing power and more reasonable statistical methods should be stressed to improve gene correlation analysis.

## Data Availability

The datasets presented in this study can be found in online repositories. The names of the repository/repositories and accession number(s) can be found in the article/Supplementary Material.
