# Supplementary figures and images for: Identification of Survival-Associated Hub Genes in Pancreatic Adenocarcinoma Based on WGCNA
Source: Front Genet. 2022 Jan 3;12:814798. doi: 10.3389/fgene.2021.814798 (PMC8762281; doi:10.3389/fgene.2021.814798)

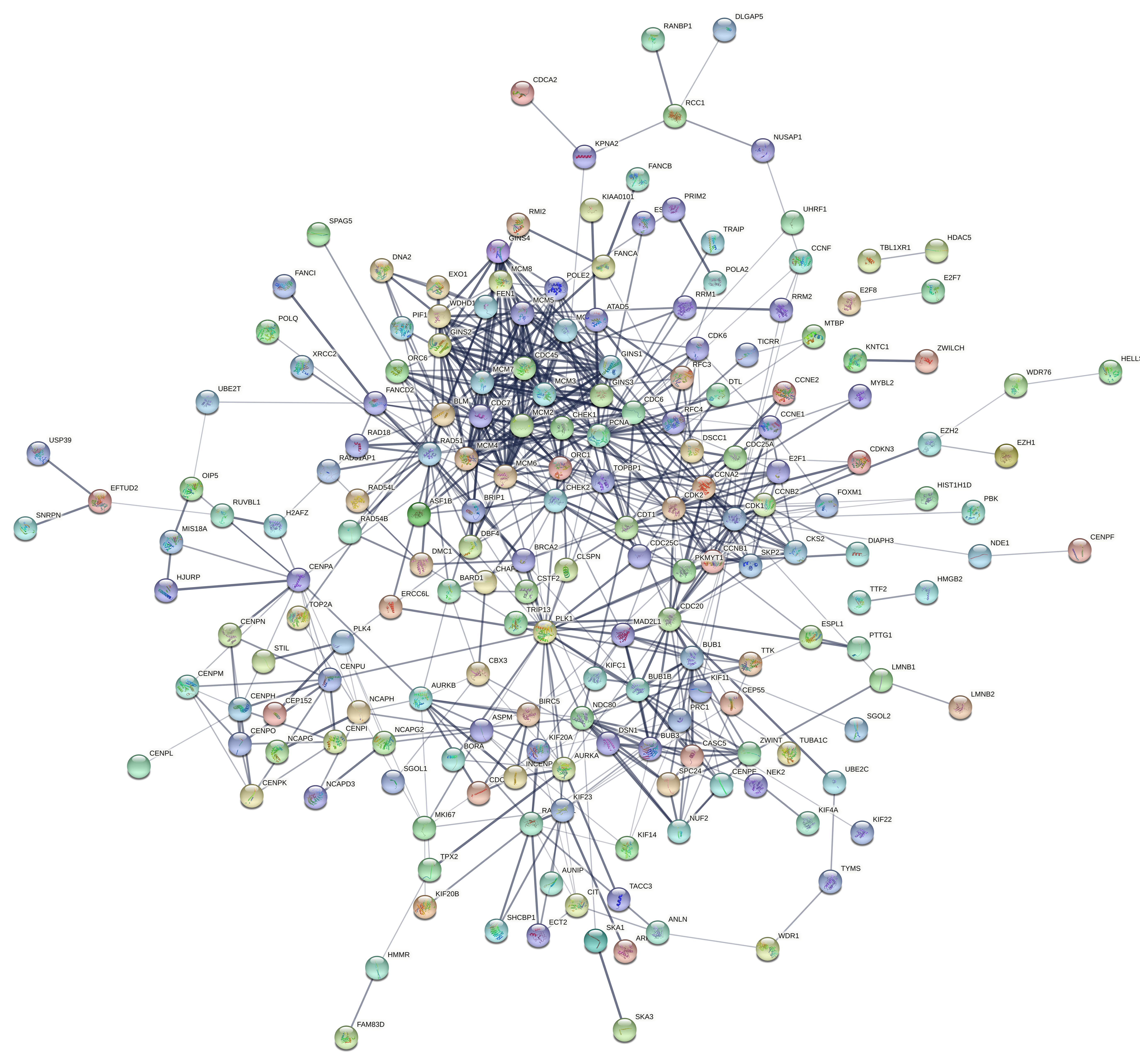

Supplement: Supplementary file 2 [file Image2.png]

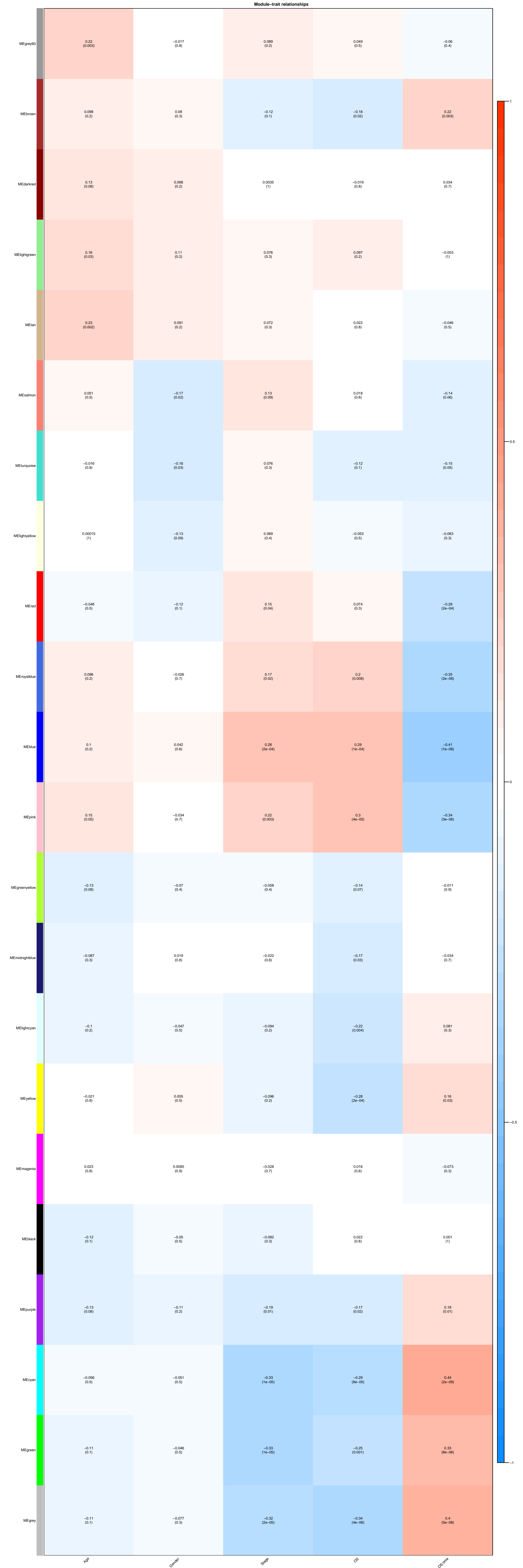

Supplement: Supplementary file 3 [file Image1.pdf]
